# Speeding up eQTL scans in the BXD population using GPUs

**DOI:** 10.1093/g3journal/jkab254

**Published:** 2021-08-16

**Authors:** Chelsea Trotter, Hyeonju Kim, Gregory Farage, Pjotr Prins, Robert W Williams, Karl W Broman, Śaunak Sen

**Affiliations:** 1 Department of Preventive Medicine, University of Tennessee Health Science Center, Memphis, TN 38163, USA; 2 Department of Genetics, Genomics and Informatics, University of Tennessee Health Science Center, Memphis, TN 38163, USA; 3 Department of Biostatistics and Medical Informatics, University of Wisconsin-Madison, Madison, WI 53706, USA

**Keywords:** linear model, genome scan, BXD, GPU

## Abstract

The BXD family of mouse strains are an important reference population for systems biology and genetics that have been fully sequenced and deeply phenotyped. To facilitate interactive use of genotype–phenotype relations using many massive omics data sets for this and other segregating populations, we have developed new algorithms and code that enable near-real-time whole-genome quantitative trait locus (QTL) scans for up to one million traits. By using easily parallelizable operations including matrix multiplication, vectorized operations, and element-wise operations, our method is more than 700 times faster than a R/qtl linear model genome scan using 16 threads. We used parallelization of different CPU threads as well as GPUs. We found that the speed advantage of GPUs is dependent on problem size and shape (the number of cases, number of genotypes, and number of traits). Our approach is ideal for interactive web services, such as GeneNetwork.org that need to display results in real-time. Our implementation is available as the Julia language package LiteQTL at https://github.com/senresearch/LiteQTL.jl.

## Introduction

The BXD family is a deeply phenotyped cohort of recombinant inbred mouse strains that have been used since the early 1970s for genetic analysis and quantitative trait locus (QTL) mapping (Ashbrook *et al.* 2019). For the past 20 years, they have been used in many large-scale omics and expression quantitative trait locus (eQTL) studies (Chesler *et al.* 2004). There are currently 150 fully inbred BXD strains, all of which have been repeatedly genotyped at many thousands of SNPs and SSLPs. Thus any new omics data can be immediately used for quantitative expression trait locus (QTL or eQTL) mapping and for association analyses with previously collected phenotypes. For omic data sets collected using high-throughput technologies, additional analyses, such as transcriptional network construction or causal mediation analyses, are also practical.

The open-source GeneNetwork web service (www.genenetwork.org) (Chesler *et al.* 2004; Sloan *et al.* 2016; Mulligan *et al.* 2017) facilitates systems genetics and mapping by providing a searchable and exportable database of phenotypes and genotypes for a variety of organisms (including mouse, rat, and *Arabidopsis*). It also provides a suite of interactive tools for browsing data, generating QTL maps, correlational analyses, network construction, and genome browsing. We wanted to develop a backend for web services such as GeneNetwork to perform real-time eQTL analysis of tens of thousands of omics traits using key populations such as the BXDs.

To perform eQTL scans in the BXD family, one has to perform as many genome scans as there are phenotypes. This can be done in an “embarrassingly parallel” fashion by using standard algorithms for QTL analysis, such as those employed by R/qtl (Broman *et al.* 2003). In practice, this is too slow, and speedup tricks are useful. For example, by using the Haley-Knott algorithm (Haley and Knott 1992) using genotype probabilities instead of the Expectation-Maximization (EM) algorithm (Lander and Botstein 1989), and processing phenotypes with the same missing data pattern in batches, instead of processing each phenotype individually, substantial speedups are possible. This is a well-known trick and is used by R/qtl. In addition, if only additive effects are tested, or if the population has only two genotype categories (as in a backcross or recombinant inbred line), then matrix multiplication can be used to perform Haley-Knott regression (Shabalin 2012).

Processing large data sets have been a challenge for genome scans. We have benefited from Moore’s Law for decades, but the central processing unit (CPU) technology is approaching the physical limits of packing transistors. Graphical processing units (GPUs), originally used as an image processing component of a computer, have shown some compelling results to accelerate computation in various fields. General purpose graphics processor units (GPGPUs) became popular in the early 2000s because of their ability to natively handle matrix and vector operations. Such power is attractive to the scientific computing community. Zhang *et al.* (2015) used GPUs to simultaneously dissect various genetic effects with a mixed linear model. Chapuis *et al.* (2013) utilized GPU to offset heavy computation to deploy various ways for a more precise calculation of a QTL detection threshold. By using GPU-backed machine learning libraries such as PyTorch, Taylor-Weiner *et al.* (2019) re-implemented QTL mapping and Bayesian nonnegative matrix factorization and reported achieving greater than 200-fold speedup compared to CPU versions. The ease of using such libraries has motivated the development of new methods for genomic research.

We build upon these efforts to perform real-time eQTL scans for the BXD family using both CPU and GPU systems. Since programming for GPUs is often nontrivial, needing the use of low-level languages such as C++, we used the Julia programming language (Bezanson *et al.* 2017) that offers GPU programming capabilities while retaining the simplicity of a high-level language such as R or MATLAB. Finally, since most phenotype-marker associations are null, we examined the impact of storage precision, and of only returning the highest association (log of odds, LOD) score for each trait instead of a matrix of LOD scores for every pair of marker and phenotypes (returning the maximum LOD per trait speeds computation by reducing output size). We have achieved computing speeds to the extent that almost all response latency is now related to data transfer and browser display, rather than the computation. This makes real-time eQTL scans practical for the BXDs and many other similar populations.

## Materials and methods

We used two BXD transcriptome datasets for developing and refining our methods. All data were downloaded from GeneNetwork (see *Data Availability* section). The genotype file includes 7321 markers by 198 BXD strains; the spleen dataset has data for 79 BXD strains and for 35,556 transcripts while the hippocampus dataset has data for 70 BXD strains and 1,236,087 probe sets. Data cleaning and wrangling were performed using R/qtl (Broman *et al.* 2003) and R/qtl2 (Broman *et al.* 2019).

### Linear model

Let *y_i_* denote a vector for the *i*-th expression trait (i=1,…,m) for *n* individuals. We define a univariate linear model as follows:

$$y_{i}=X_{j}\beta_{j}+\epsilon_{i},\;\epsilon_{i}∼N(0,\sigma_{i}^{2}I),$$

where $X_{j}$ is a matrix including the intercept and the *j*-th candidate genetic marker (j=1,…,p) without covariate(s), $\beta_{j}$ is a vector of the *j*-th eQTL effects, and $\epsilon_{i}$ is random error distributed as $N(0,\sigma_{i}^{2}I)$. We assume to be interested in one-df tests as would be the case for genome scans in the BXDs. Suppose $RSS_{0i}$ is the residual sum of squares under the null hypothesis of no eQTL, and $RSS_{1ij}$ is the residual sum of squares under the alternative of existing eQTL at the *i*-th trait and the *j*-th genetic marker. Then, the *LOD_ij_* score for a one-df test can be written as:

$$\begin{array}{c} LOD_{ij}=\frac{n}{2}\;{\text{log}}_{10}\left(\frac{RSS_{0i}}{RSS_{1ij}}\right)\\ =\frac{n}{2}\;{\text{log}}_{10}\left(1-r_{ij}^{2}\right), \end{array}$$

where *r_ij_* is the correlation between the *i*-th expression trait and *j*-th marker. If $Y^{*}$ and $G^{*}$ are respectively standardized trait (*Y*) and genotype (*G*) matrices (*i.e.*, with the columns centered and scaled to have mean 0 and variance 1), then the correlation matrix is simply

$$R=\frac{1}{n}Y^{*\prime }G^{*}.$$

**Figure 1:**
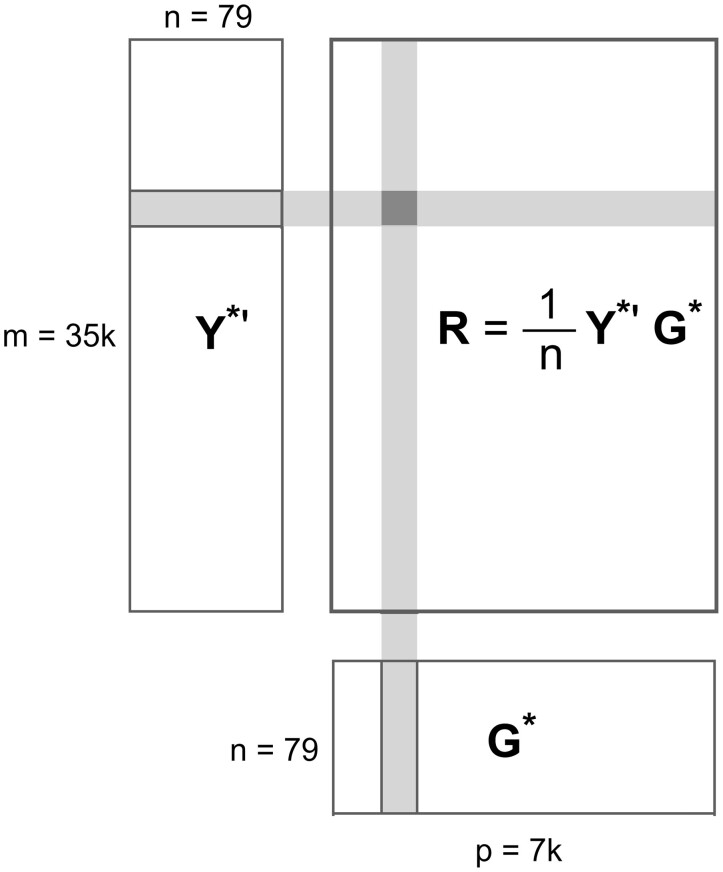
Schematic of data and correlation calculation: $Y^{*}$ is a standardized expression phenotype matrix, $G^{*}$ is a standardized genotype matrix, and *R* is a correlation matrix. An entry in *R* (shaded dark gray) is obtained by summing the product of the entries of the corresponding row of $Y^{*\prime }$ and the corresponding column of $G^{*}$ (both shaded light gray). The matrix of LOD scores is an element-wise function of the correlation matrix.

Since matrix multiplication is a parallelizable operation for which optimized routines are available, this formula is very attractive for bulk calculation of LOD scores. The formula can be extended for LOD scores adjusted by covariates. The idea is to project genetic markers and gene expressions onto the space orthogonal to the covariates and to compute the corresponding correlation matrix just as we did for the case without covariates. In other words, let *Z* be a matrix of covariates including intercept. The projection orthogonal to the covariate space is then $P=I-Z{\left(Z^{\prime }Z\right)}^{-1}Z\prime$. The genotype matrix (*G*) and gene expressions (*Y*) are now transformed into *G_z_* = *PG*, *Y _z_* = *PY*, respectively. This is the same as calculating the residuals after regressing on *Z*. Standardization followed by multiplication of the matrices yields the correlation matrix ($R_{z}=\frac{1}{n}Y_{z}^{*\prime }G_{z}^{*}$) just as shown above. Figure 1 gives a visual representation of the matrix multiplication.

### Acceleration techniques

While it is true that many programs can achieve 10- or even 100-fold speedup by utilizing GPUs, the difference needs to be examined with care. Sometimes, a reported CPU time is using a single thread, and multithreaded CPU time may bring the performance gap between CPU and GPU narrower than claimed. Also depending on the library chosen for the CPU, the speed might vary depending on whether the library is optimized for such computation or hardware. We believe for a fair comparison, both CPU and GPU functions should be optimized at maximum performance and should account for all necessary overhead. The following section explains optimization efforts of CPU and GPU functions.

#### Multithreaded CPU operations

Our goal was to build a backend for web services such as GeneNetwork that allow researchers to interact with data in real-time. That requires that the genome scan finish within seconds. To bring out the best performance of CPUs, we use multi-threaded operations whenever possible. Julia (Bezanson *et al.* 2017), our choice of programming language, provides simple yet safe syntax for multi-threading. It is done by adding the Threads.@thread macro to indicate to Julia that the following for loop is the multi-threaded region. The Threads.nthreads() function shows the number of threads in Julia, and the default number of threads we use is 16.

#### GPU operations

Originally used in graphics, GPUs have taken off as a general computing device in recent years because they provide a massive number of cores at a lower price range and because of the availability of fast GPGPU libraries such as CUDA (Compute Unified Device Architecture) and OpenCL (Open Computing Language). Based on our profiling results, the time consuming parts of our genome scan method are matrix multiplication and element-wise operations. Both are amenable to GPU heterogeneous computing architecture since they have no data race conditions (where processes depend on each other’s results) and low data dependencies. However, the GPU also has its own limitations. To truly utilize the maximum computing power of GPUs, one needs to think creatively to work around those limitations. For example, during our experiments, we found that memory transfer between host and device is really slow. Profiling the result shows that 98% of total genome scan time is spent on memory transfer. This is because the size of the output matrix (of genome scan LOD scores) is much larger than the size of the input matrices (of genotype probabilities and phenotypes). To cope with this capacity constraint, instead of offloading the entire correlation matrix, we use the GPU to calculate the maximum LOD score of each expression trait and output the maximum. The output matrix is now much smaller, and the memory transfer times are reduced. This allows us to identify transcripts with at least one eQTL and speeds up the computation substantially.

#### Matrix and vectorized operations

Since our algorithm largely depends on matrix operations, it is natural to find the fastest way to achieve the best result regardless of computing platforms. There are various matrix libraries available for CPU, such as gslBLAS and OpenBLAS (Wang *et al.* 2013). They target different hardware or use various techniques to get optimal results. Multi-threaded matrix multiplication is the default in OpenBLAS, and does not require extra coding effort to parallelize the CPU version of matrix multiplication. We, therefore, chose OpenBLAS as our CPU computing library.

Matrix multiplication and element-wise operations are algorithmically free of data and function dependency, so that they are amenable to GPU’s parallel computing power. Julia provides various packages for GPU including CUDA (Nickolls *et al.* 2008) bindings. Our chosen hardware for GPU is from Nvidia, which requires its proprietary library, CUDA, which is mature and well-recognized in the scientific computing community. For matrix operations on GPU, we used the cuBLAS library (Nvidia 2021a), which provides a fast GPU implementation of BLAS (Basic Linear Algebra Subprograms) from Nvidia.

We investigated the effect of matrix shape on the speedup in addition to the effect of using the GPU for multiplication. Of course, most of the time, one cannot pick the size and shape of data in a matrix form, but such information would help researchers as a rough guidance of whether it is worth considering the GPU option before investing programming efforts for GPUs. We ran matrix multiplication with different shapes of matrices and compared the runtime of CPU and GPU. CPU time is measured by matrix multiplication from the OpenBLAS library using 16 threads. GPU time includes all overhead of using GPU, which involves device launch, data transfer, and all necessary API calls. In order to make a fair comparison between CPU and GPU, we needed to use maximum strength of both and include all necessary cost.

The experiment setup was to multiply two input matrices, A(m × n), and B(n × p), and produce an output matrix C (m × p). The range of *m*, *n*, and *p* is between 2^4^ and 2^17^ in powers of 2. We compared the result when the size of input and output (I/O size) matrices, in total, was between 11 and 16 GB.

#### Single precision

Precision means the smallest difference between two representable numbers. Floating point numbers, in scientific computing, are usually stored in double precision. Double precision floating point numbers take up 8 bytes in memory while single-precision numbers take up 4 bytes. In addition to the difference in storage size, the speed for calculation using single and double precision also varies by hardware. For example, the GPU throughput (the number of floating point calculation per second, measured in FLOPS) for double precision is 1/32 of single precision on a Nvidia GTX 1050 GPU, and 1/4 on a Nvidia Tesla K80. Thus, single-precision brings multiple benefits when precision is not the primary concern.

#### Julia language

Although a programming language cannot really be classified as an optimization technique, the choice of programming language can affect run time as well as development time. We chose Julia, an interpreted language with a just-in-time compiler, that provides fast runtimes approaching compiled languages such as C/C++ with the development ease of interpreted languages such as Python or R. Julia also has packages that make it easy to use GPUs, and even program some GPU kernels purely in Julia without resorting to C or C++.

#### Comparison with tensorQTL

TensorQTL (Taylor-Weiner *et al.* 2019) is a GPU-enabled QTL mapper that reported approximately 200- to 300-fold faster QTL mapping compared to CPU-based implementations. We compared our implementation to tensorQTL noting that our implementation was primarily developed for experimental cross populations (such as the BXD population) while tensorQTL was optimized for outbred populations such as humans. We used the open-source code of tensorQTL to time key parts of the computational pipelines: data transfer (getting data to and from device), core computation (eQTL scans), post processing (other related cost to generate a meaningful output, such as calculating *P*-values, concatenating dataframes, adding additional genotype and phenotype information, type conversions, and so on), and the total elapsed time. No alterations to the tensorQTL algorithm were made. For each program, we timed two versions, one that returned the full matrix of LOD scores (LiteQTL) or *P*-values (tensorQTL), and the other that returned a filtered set: maximum LOD score for each transcript, (LiteQTL) or all *P*-values lower than $10^{-5}$ (tensorQTL). Both programs filtered for MAF (minor allele frequency).

Runtimes were based on the mean of 10 runs on the same hardware using the GEUVADIS dataset provided by tensorQTL. We analyzed 19,836 traits and 20,000 genotypes on chromosome 9 to ensure that the data fitted in the GPU. For a more detailed description, please see the Supplementary material.

### Platform

Our platform for computation:

Hardware:

- CPU: Intel(R) Xeon(R) Gold 6148 CPUs @ 2.40 GHz; 80 cores, 187 GB
- GPU: Tesla V100-PCIE-16 GB; 5120 CUDA cores

Software:

- OS: Debian GNU/Linux 10
- Programming environment: Julia v1.5
- Libraries: CUDA v10.1 and cuBLAS; OpenBLAS
- Profilers: Julia Profiler; nvprof
- tensorQTL v1.0.4; LiteQTL v0.2.0; R/qtl v1.47

### Software availability

We have created a Julia package for performing the computations mentioned in this study. It can be installed from the Julia command line as the LiteQTL package from the Julia General registry. The source code for this package is publicly available on Github (https://github.com/senresearch/LiteQTL.jl) The repository contains an example directory with a Jupyter notebook that shows how to compute eQTL scans for the BXD spleen dataset and make a plot of the eQTLs. To benefit from the GPU options, users will need an Nvidia GPU in their machine and have the Julia executable, openBLAS, and CUDA libraries installed. The Supplementary material accompanying this study can be found at https://github.com/senresearch/LiteQTL-G3-supplement.

## Results

**Figure 2:**
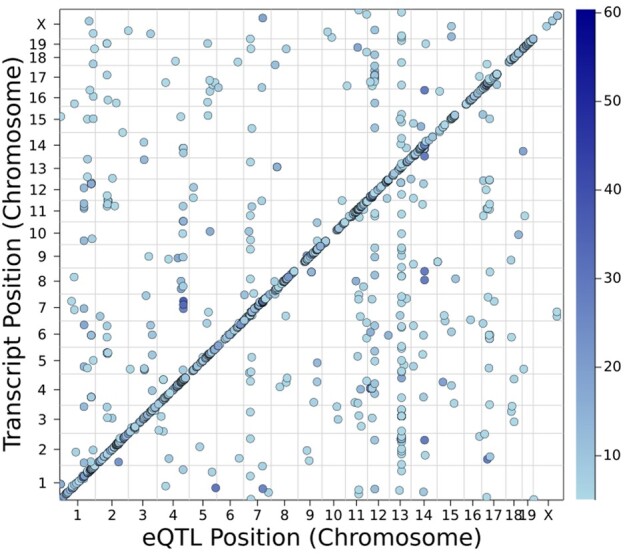
Distribution of eQTL across genome in the BXD spleen dataset. On the vertical axis, we plot the physcal location of the cognate gene for a transcript; transcripts without a good match to a known gene are not shown. On the horizontal axis, we plot the location of the marker with the highest LOD score for each transcript provided it exceeded 5.

We performed eQTL scans in the spleen dataset (36K traits) in 0.06 seconds, and for the hippocampus dataset (1.2M traits) in 2.82 s with our hardware. Below, we show how the algorithm choice, CPU/GPU, precision, and programming language impacted our results. For the spleen dataset, Figure 2 shows the position of the strongest eQTL for transcripts with a maximum LOD score greater than 5 against the physical position of the cognate gene, if known, of the transcripts. Of the 35,554 transcripts, 2057 (5.8%) had a maximum LOD exceeding 5.

### Effect of matrix shape on matrix multiplication speed in GPU

**Figure 3:**
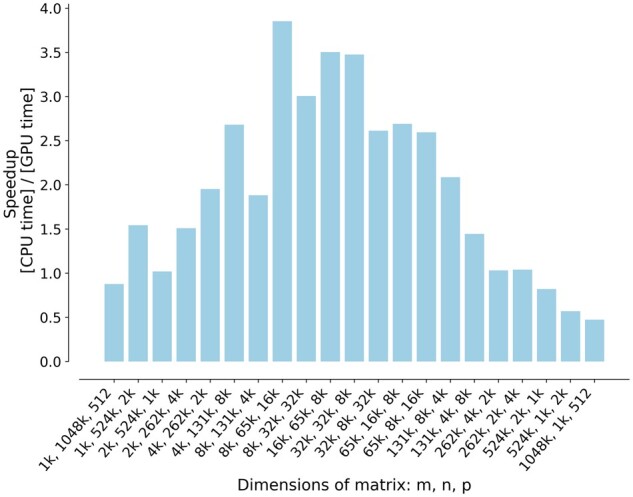
Variation of GPU *vs* CPU speedup with matrix shape for calculating C=AB. The matrix with more squared shapes gains relatively better speedup compared to the ones with long or wide shapes. The *x*-axis shows *m*, *n*, *p*, where dimension of *A* is m × n, n × p for *B*, and m × p for *C*.

The result of our experiment is shown in Figure 3. The *x*-axis of Figure 3 is the dimensions of matrix. The two ends of *x*-axis represent matrices with slender or wider matrices, while the middle of *x*-axis represents matrices closer to square shape. The *y*-axis is the speedup of GPU compared with CPU on a linear scale. From this figure, we see that matrices whose shapes are relatively closer to square get better speedups from GPU. Matrix multiplication is up to 3.85 times faster on GPU than on 16 threaded CPU on our hardware.

### Benefit of customized algorithm for eQTL scans

R/qtl is a general-purpose QTL mapping program. To provide a baseline for our approach customized for eQTL scans, we compared runtimes to R/qtl. The timing of our method and R/qtl is shown in Table 1. By simplifying the genome scan process, using matrix multiplication, and returning the maximum LOD, we observed a significant speedup. For the spleen data, our method was 48 times faster (0.83 s for LiteQTL on CPU only *vs* 40.02 s for R/qtl). For the hippocampus data the speedup was 125 times (16.57 s on CPU only *vs* 2070.71 s for R/qtl), and 734.2 times if R/qtl is compared with LiteQLTL’s CPU & GPU option.

**Table 1:** eQTL scan runtimes for R/qtl and LiteQTL

Dataset	Precision	LiteQTL			R/qtl	LiteQTL speedup *vs* R/qtl	
		CPU only	CPU and GPU	GPU speedup		CPU only	CPU and GPU
Spleen (35K traits)	Single	0.56 s	0.04 s	14.0x	—	—	—
	Double	0.83 s	0.06 s	13.8x	40.02 s	48.2x	667.0x
Hippocampus (1.2M traits)	Single	12.36 s	1.66 s	7.4x	—	—	—
	Double	16.57 s	2.82 s	5.4x	2070.71 s	124.9x	734.2x

We show the time taken in seconds to perform eQTL genome scans with LiteQTL and R/qtl. LiteQTL times are shown by precision (single *vs* double), and whether the GPU was used or not. The GPU speedup column computes the speedup for LiteQTL using the CPU and GPU *vs* using the CPU only. LiteQTL was used with the maximum LOD output option to reduce data transfer time.

### Benefit of using GPU

In parallel computing, Amdahl’s law indicates the theoretical maximum speedup that could be attained when improving a particular part of a program. For example, if a program takes 10 min for a serial processor and a function that takes nine of those 10 mins can be parallelized, then the theoretical speedup, no matter how many processors are used, cannot be more than 10 times because the minimum execution time of this program is 1 min. Therefore, profiling the entire genome scan process is a prerequisite for optimization. Often, profiling would consider space and time complexity. Our primary concern is the time taken by each function, and therefore only timing information is considered in our profiling. We used Julia’s built-in sampling profiler to find our target functions for GPU because it is less intrusive than the other profiling methods.

The genome scan process includes the following steps:

- Calculate standardized matrices ($\frac{1}{\sqrt{n}}G^{*},\;\frac{1}{\sqrt{n}}Y^{*}$) for input matrices (*G*, *Y*)
- Get a correlation matrix (*R*) by multiplying the standardized matrices
- Calculate LOD scores

Our profiling result shows that the second and third steps take up over 90% of the computation time and involve parallelizable matrix operations. Hence, they are our candidates for GPU acceleration.

We used a GPU profiler *nvprof* (Nvidia 2021c) to identify bottleneck of GPU. The results indicate that 98% of the GPU running time is spent on data transfer from GPU to CPU (device to host). As shown in Figure 1, the input matrices Y’ and G are small compared with the output matrix R. For the BXD spleen dataset, Y’ matrix is 17 MB, G matrix is 21 MB, but R matrix is about 4GB. Data offloading is the main bottleneck for our GPU implementation.

To overcome this limitation of GPUs, we only offload the maximum of LOD score of every phenotype since that is the primary interest for initial exploration. Finding the maximum is highly parallelizable, can utilize GPU’s massive cores, and reduces the amount of data that needs to be transferred back to host.

The timing shown in Table 1 is the total execution time and necessary overhead for genome scan. We ran the genome scan process 10 times and chose the median to remove the randomness of each run and warm-up time of GPUs.

In Table 1, CPU & GPU implementation gains 5–14 times speedup compared to CPU only. The former took only 0.83 and 16.57 s for spleen and hippocampus dataset, while the latter took 0.06, and 2.82 s respectively. Our algorithm exploits parallelism in two ways, by simplifying the genome scan process to matrix multiplication, and by getting the maximum LOD score of each phenotype. Such arrangement is ideal for GPU processing: maximum parallelization for computation while minimizing data input and output.

### Benefit of using single precision

Table 1 also shows the execution time using single and double precision. In all cases, genome scans run faster using single precision than using double precision. The speedup are more appreciable in the larger, hippocampus dataset compared to the spleen dataset. Using single precision provides benefits in three aspects: memory storage, data transfer, and arithmetic calculation.

### Benefit of using Julia

In our explorations of matrix multiplication, Julia’s speed is comparable to C/C++ (results not shown). However, the low learning curve, clean syntax, as well as support for GPU programming libraries such as CUDAnative (Besard *et al.* 2018) reduce programming effort relative to C/C++. Compared with writing GPU functions in C/C++, writing in Julia is cleaner and easier because it requires much less boilerplate code. Below are some example code snippets. The first example shows how to call cuBLAS from Julia, and the second example shows how to write a custom kernel in Julia. To respect page limits, we will not show the corresponding C code. An example of using cuBLAS with C can be found online (Nvidia 2021b).

**## Example 1:**

using CUDA

A = rand(1000,1000)

B = rand(1000,1000)

**# Data transfer from CPU to GPU**

d_a=CuArray(A)

d_b=CuArray(B)

**# GPU matrix multiplication calling CuBLAS library**

d_c = CUDA.CUBLAS.gemm('T', ‘N’, d_a, d_b);

**# Data Transfer from GPU to CPU**

C=collect(d_c)

**## Example 2:**

**# Custom kernel for matrix element-wise calculation**

function log_kernel(data, MAX)

**# calculating GPU thread ID**

i=(blockIdx().x-1)*blockDim().x+threadIdx().x

**# Check thread ID is in bound.**

if(i < MAX + 1)

**# Call log function on GPU**

data[i]=CUDAnative.log(data[i])

end

return

end

**# initialize and transfer data to GPU*.*

MAX= 64000

d_data=CuArray(rand(MAX))

**# Launching GPU**

d_res = @cuda blocks = 1000threads = 64

log_kernel(d_data, MAX)

**# Transfer result back to CPU**

res=collect(d_res)

### Comparison with tensorQTL

Table 2 shows the comparison of runtimes broken down by time taken for data transfer, core computation, and post processing. The main finding is that the data transfer and core computation take about the same time for tensorQTL and LiteQTL for both CPU and GPU. Both program timings indicate a speedup factor of 20 times for the core computation of the full matrix. For the filtered version, the GPU was about 56 times faster than CPU only for tensorQTL and about 18 times faster for LiteQTL.

**Table 2:** Timing comparison between tensorQTL and LiteQTL: Times are averaged over 10 runs and expressed in seconds

	tensorQTL				LiteQTL			
	Full matrix		Filtered *P*-value		Full matrix		Filtered max	
	CPU only	CPU and GPU	CPU only	CPU and GPU	CPU only	CPU and GPU	CPU only	CPU and GPU
Data transfer	0.015	0.561	0.018	0.069	0.000	0.660	0.000	0.020
Core computation	0.940	0.055	1.601	0.029	1.022	0.054	0.536	0.030
Post processing	9.865	8.060	0.777	0.719	0.000	0.785	0.000	0.030
Elapsed	10.820	8.676	2.396	0.817	1.022	1.499	0.536	0.080

Full matrix timings are done without any filtering threshold. Filtering threshold is different for tensorQTL and LiteQTL. For tensorQTL, the MAF (Minor Allele Frequency) threshold is 0.05, and the *P*-value threshold is $10^{-5}$. For LiteQTL, the MAF threshold is 0.05, and the maximum LOD score for each transcript. The main conclusion is that the core computation and data transfer between tensorQTL and LiteQTL is very similar. The difference lies in post processing, which varies a lot depending on filtering threshold, and user-defined output.

Depending on user-defined options, the output may need further processing after the core computation. This post processing is responsible for the main differences in elapsed time. Because it depends on user options, both programs exclude that time in calculating the GPU speedup. In our tests, the elapsed time for tensorQTL was longer than for LiteQTL.

## Discussion

We examined the effectiveness of using GPUs for speeding up eQTL scans in the BXD family of recombinant inbred lines. We are able to run genome scans for the spleen data (36K traits) in 0.06 s and for the hippocampus data (1.2M traits) in 2.82 s. This meets the requirements for real-time performance. Although there are additional hurdles in deploying the GeneNetwork front-end web service, this is very encouraging. Users can use our stand-alone Julia package for running eQTL scans.

For us, the GPU speedup compared to CPU implementation is best when the matrices are closer to square shapes. On our test hardware, matrix multiplication is up to 4 times faster on GPU than on 16 threaded CPUs. The exact speedups with our algorithm and software will depend on the hardware configurations.

The 200–300 times speedup reported by Taylor-Weiner *et al.* (2019) does not include data transfer, and for this specific speedup, reported CPU time is single-threaded. The supplement of Taylor-Weiner *et al.* (2019) reported the speedup when GPU data transfer is included, and that brought it down in the range of 10–100. This is the same as our observation. Data transfer time needs to be included when reporting GPU time, since this is a cost associated with using GPU. Sometimes, it is a very significant cost. When LiteQTL returns the full matrix of LOD scores, the data transfer time amounts to more than 90% of total GPU time. It is also more realistic to report a multithreaded CPU time (16 threads or more), since the speedup for CPU from multithreading is easily achievable. These two factors shrink the gap between CPU and GPU. However, when the CPU multithreading acceleration is capped (the cost of maintaining multithread communication surpass the computation time), GPU can provide that extra boost, for time critical use cases.

Returning the maximum LOD score for each transcript reduces data size and speeds up computations substantially. However, this comes at a cost: to identify all eQTLs, one would need to run a secondary genome scan with all transcripts with at least one eQTL.

Our experience in this project indicates that the excitement about using GPUs for speeding computations needs to be tempered by the limitations of GPU. It takes significant additional development time and therefore, is most useful for high-value (deep learning) or routine-use (graphics rendering) projects. We also need to reconsider algorithms with the GPU in mind, and pay attention to the speed and size of data to be transfered to/from CPU to GPU. In many problems it is much easier to throw additional CPU cores to the problem with minimal programming than to devote effort into GPU programming. Both Taylor-Weiner *et al.* (2019) and LiteQTL attempt to address this barrier, making GPU algorithms more available to a broader range of users.

LiteQTL only supports a 1-degree freedom test currently and assumes missing data in the input data set; any missing data has to be handled in pre-processing and will add to the computation time. Currently, the LOD scores are fit using a linear model; for many problems a linear mixed model (LMM) (Lippert *et al.* 2011) is of interest. We expect to build on the current work to tackle that problem in the future.

## Funding

This work was funded by National Instiutes of Health grants P30DA044223 (P.P., R.W.W., and S.S.), R01GM123489 (C.T., H.K., P.P., R.W.W., K.W.B., and S.S.), and R01GM070683 (K.W.B. and S.S.).

## Data availability

The BXD Genotype Database (GN Accession: GN600) and two sets of transcriptome data, UTHSCAffy MoGene 1.0 ST Spleen (GN Accession: GN283) and UMUTAffy Hippocampus Exon (GN Accession: GN206) can be obtained from GeneNetwork https://GeneNetwork.org. The GEUVADIS data for tensorQTL comparison can be obtained from tensorQTL’s Github repository: https://github.com/broadinstitute/tensorqtl/blob/master/example/tensorqtl_examples.ipynb Supplemental material is available at figshare: https://doi.org/10.25387/g3.14622603.

## Conflicts of interest

The authors declare that there is no conflict of interest.
